# Supplementary material for: Prognostic Role of miR-221 and miR-222 Expression in Cancer Patients: A Systematic Review and Meta-Analysis
Source: Cancers (Basel). 2019 Jul 11;11(7):970. doi: 10.3390/cancers11070970 (PMC6678869; doi:10.3390/cancers11070970)
Supplement: Supplementary file 1 [file cancers-11-00970-s001.pdf]

cancers

MDPI

Supplementary Materials: Prognostic role of miR-221 and miR-222 expression in cancer patients: A systematic review and meta-analysis

Gloria Ravegnini, Sarah Cargnin, Giulia Sammarini, Federica Zanotti, Justo Lorenzo Bermejo, Patrizia Hrelia, Salvatore Terrazzino and Sabrina Angelini

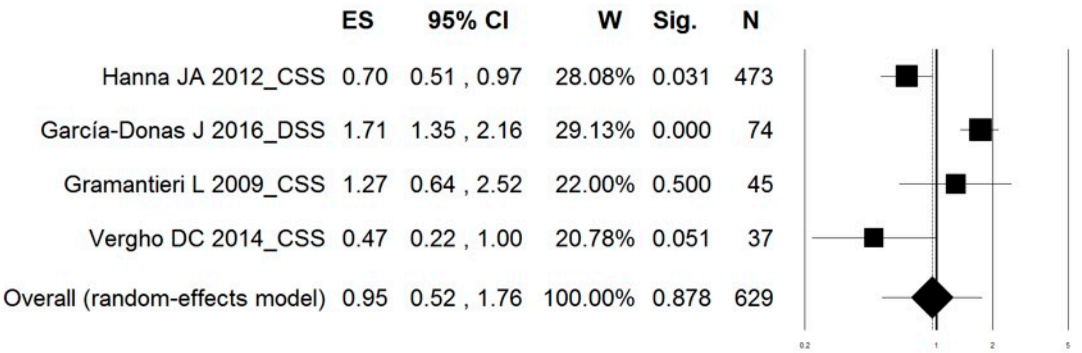

Figure S1. Forest plots for the effect of miR-221 overexpression and CSS and DSS

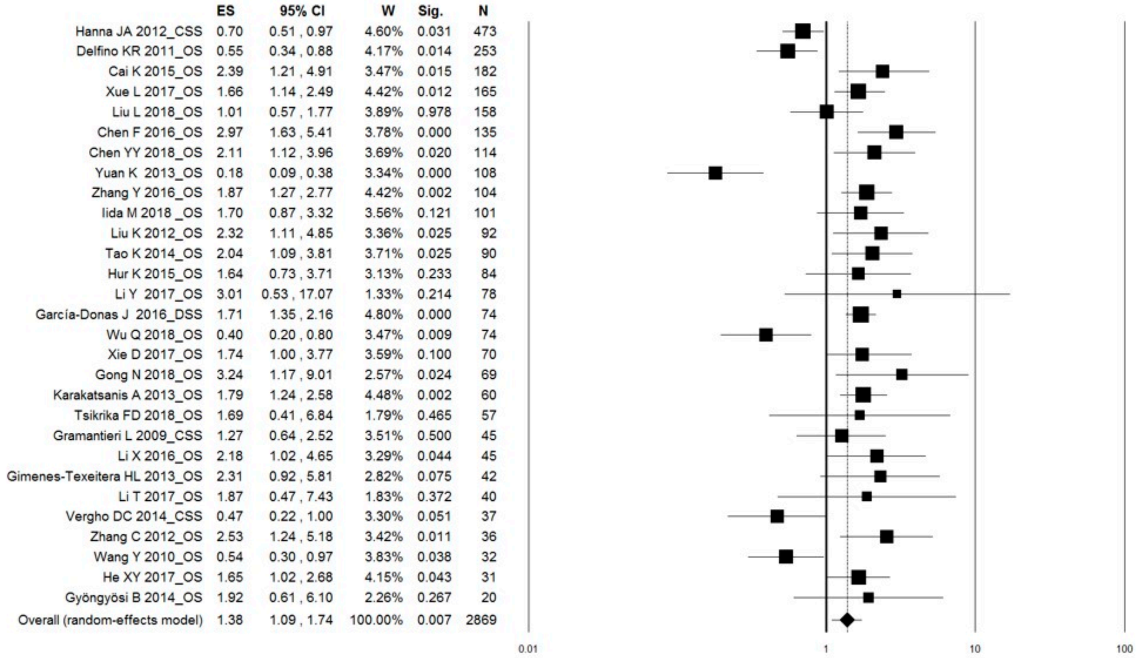

Figure S2. Forest plots for the effect of miR-221 overexpression and OS/CSS/DSS

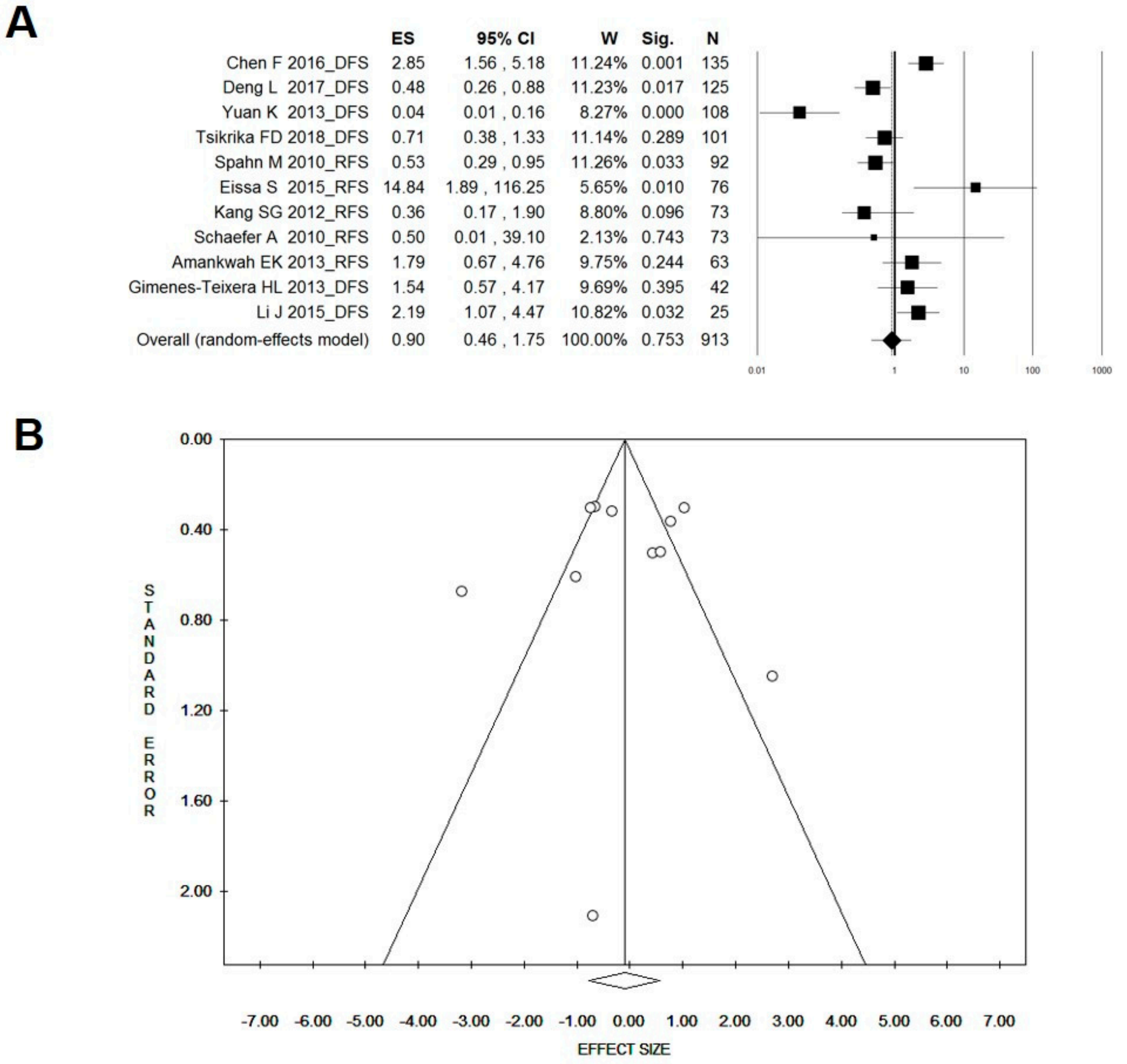

Figure S3. Forest (**A**) and Funnel (**B**) plots for the effect of miR-221 overexpression on DFS/RFS. ES, effect size (i.e., hazard ratio); W, weight; Sig, statistical significance; N, total number of cancer patients included in the survival analysis.

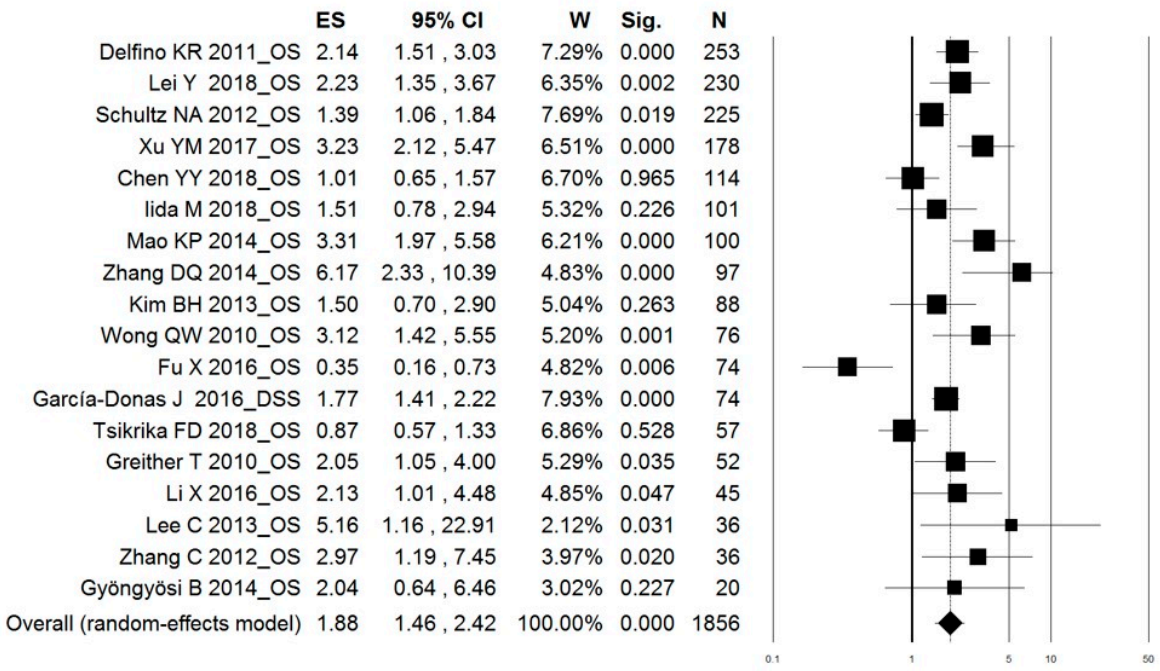

Figure S4. Forest plots for the effect of miR-222 overexpression and OS/DSS

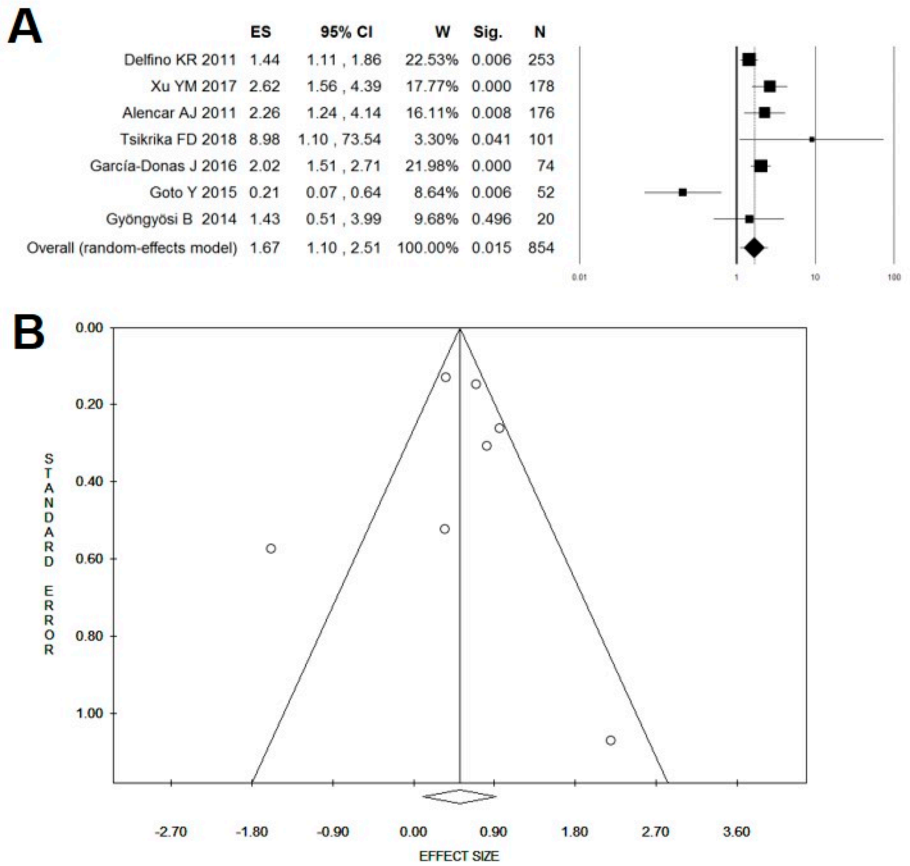

Figure S5. Forest (**A**) and Funnel (**B**) plots for the effect of miR-222 overexpression on progression-free survival. ES, effect size (i.e., hazard ratio); W, weight; Sig, statistical significance; N, total number of cancer patients included in the survival analysis.

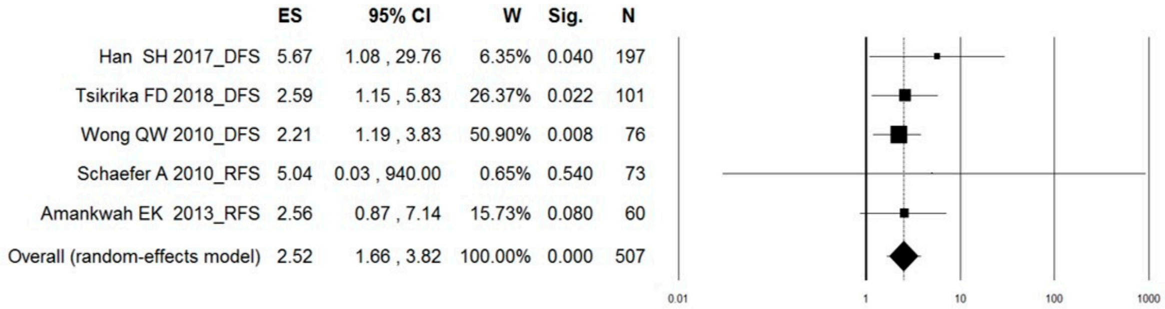

Figure S6. Forest plots for the effect of miR-222 overexpression and RFS/DFS

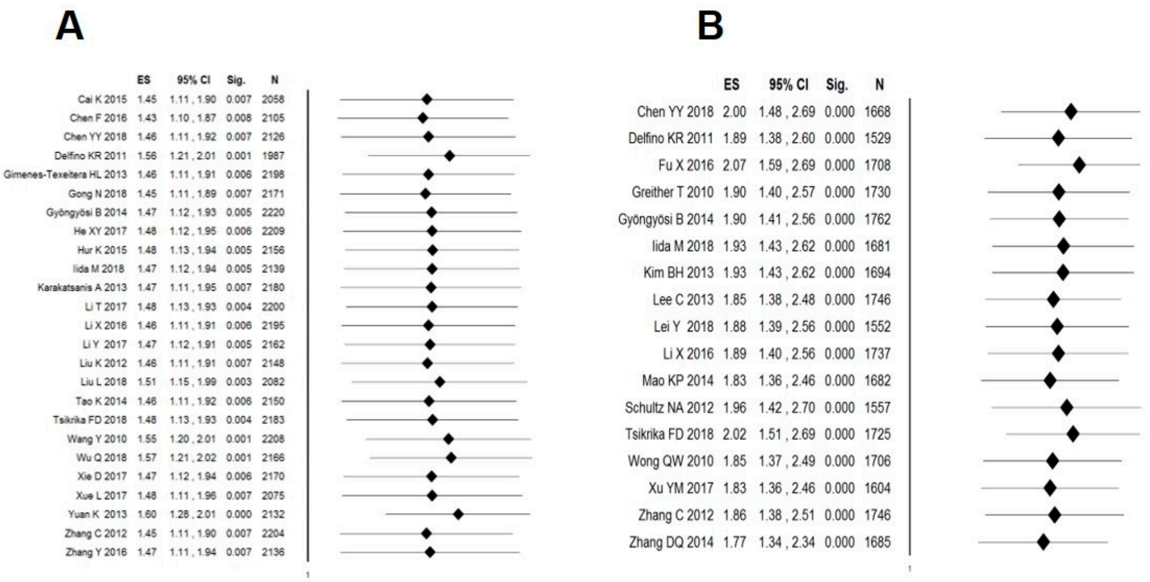

Figure S7. Sensitivity analysis of the meta-analysis for miR-221 (**A**) and miR-222 (**B**)

© 2019 by the authors. Licensee MDPI, Basel, Switzerland. This article is an open access article distributed under the terms and conditions of the Creative Commons Attribution (CC BY) license (http://creativecommons.org/licenses/by/4.0/).
